# Genomic insights into *Castanopsis carlesii* and *Castanea henryi*: flower and fruit development and evolution of NLR genes in the beech-oak family

**DOI:** 10.1186/s43897-025-00152-4

**Published:** 2025-06-04

**Authors:** Xiong-De Tu, Wen-Jun Lin, Ya-Xuan Xin, Hou-Hua Fu, Cheng-Yuan Zhou, Yi-Zhe Lin, Jun Shen, Shuai Chen, Hui Lian, Shu-Zhen Jiang, Bin Liu, Yu Li, Zi Wang, Ding-Kun Liu, Zhi-Wen Wang, Siren Lan, Ming-He Li, Zhong-Jian Liu, Shi-Pin Chen

**Affiliations:** 1 College of Forestry, Fujian Agriculture and Forestry University, Fuzhou, 350002 China; 2 Key Laboratory of National Forestry and Grassland Administration for Orchid Conservation and Utilization at College of Landscape Architecture and Art, Fujian Agriculture and Forestry University, Fuzhou, 350002 China; 3 Fujian Province Forestry Survey Planning Institute, Fuzhou, 350003 China; 4 Soil and Water Conservation Experimental Station of Fujian Province, Fuzhou, 350003 China; 5 Chongqing Engineering Research Center for Floriculture, College of Horticulture and Landscape Architecture, Southwest University, Chongqing, 400715 China; 6 College of Juncao Science and Ecology, Fujian Agriculture and Forestry University, Fuzhou, 350002 China; 7 PubBio-Tech, Wuhan, 430070 China

**Keywords:** Fagaceae, Genomics, MADS-box genes, Resistance genes, Sucrose-starch metabolism

## Abstract

The Fagaceae family, comprising over 900 species, is an essential component of Northern Hemisphere forest ecosystems. However, genomic data for tropical and subtropical genera *Castanopsis* and *Castanea* remain limited compared to the well-studied oak. Here, we present chromosome-level genome assemblies of *Castanopsis carlesii* and *Castanea henryi*, with assembled genome sizes of 927.24 Mb (N50 = 1.57 Mb) and 780.10 Mb (N50 = 1.07 Mb), respectively, and repetitive sequence contents of 45.79% and 44.88%. Comparative genomic analysis revealed that the estimated divergence time between *Castanopsis* and *Castanea* was determined to be 48.3 Mya and provided evidence that both genera experienced only one of the ancient whole genome triplication event (γ event) shared with most eudicots. The development of *C. carlesii* flower bracts and cupules was controlled by A- and E-class genes, suggesting that the cupules may originate from the bracts. Additionally, genes involved in sucrose and starch metabolism genes played distinct roles during *C. carlesii* fruit development. The amplification of the nucleotide-binding leucine-rich repeat (NLR) gene family in Fagaceae exhibited similarities, indicating that this expansion may be an adaptation to similar environmental pressures. This study provides valuable genomic resources for Asian Fagaceae and enhances our understanding of Fagaceae evolution.

**Supplementary Information:**

The online version contains supplementary material available at 10.1186/s43897-025-00152-4.

## Core

We have assembled chromosome‑level genomes of *Castanopsis carlesii* and *Castanea henryi* elucidated the genomic and NLR genes evolution of Fagaceae, and conducted studies on the development of *C. carlesii* fruits and flowers. Our study lays the foundation for a better understanding of the structure and function of the *Castanopsis* and *Castanea* genomes.

## Gene and accession numbers

The genome sequencing data and transcriptome sequencing data have been deposited at the National Genomics Data Center (NGDC, https://ngdc.cncb.ac.cn) under accession number PRJCA021809 and PRJCA021810.

## Introduction

Fagaceae, commonly known as the beech-oak family, belongs to the order Fagales of rosids. Fagales includes seven families, with Nothofagaceae as the basal taxon and Fagaceae as the sister group to other families (Li et al. 2004). Fagaceae comprises over 900 species in eight genera (Zhou et al. 2022; POWO 2024), and is primarily distributed in temperate, subtropical, and tropical forests throughout the Northern Hemisphere (Kremer et al. 2010). The discovery of the early Eocene infructescence of *Castanopsis* and the co-occurring leaves in the 52-million-year-old Laguna del Hunco flora of southern Argentina expanded the historical distribution of the beech-oak family to the Southern Hemisphere (Wilf et al. 2019). Fagaceae species are notable for their diverse fruit shapes and distinctive cupules, which remain evolutionarily enigmatic (Forman 1966). However, the evolution of cupules in Fagaceae and Nothofagaceae remains unclear. Fagaceae is of ecological importance, with three species-rich genera, namely stone oaks (*Lithocarpus*), tropical chestnuts (*Castanopsis*), and *Cyclobalanopsis* (synonym of *Quercus* L.), which make significant contributions to forest ecosystems in Southeast and South Asia (Deng et al. 2018; Yang et al. 2018). Fagaceae is also economically significant as woody crops such as chestnuts, which contain starch-rich fruit, and as valuable wood species such as oak. To date, genomes of more than ten *Quercus* species have been assembled. Research on *Q. robur* has shown that the expansion of resistance-related (R) genes is critical for the survival of long-lived trees (Plomion et al. 2018). However, research on the Asian species *Q. mongolica* suggest significant contractions in these genes, potentially due to reduced pathogens pressures in East Asian (Ai et al. 2022).

In contrast, genomic data for *Castanopsis* and *Castanea* species remain relatively scarce. The genome of the first *Castanopsis* species, *C. tibetana*, was published in 2021, with a genome size of 878.6 Mb and 54.3% repetitive sequences (Sun et al. 2022). Subsequently, the genome of the *C. hystrix* species was published, revealing that the gene family of *Castanopsis* plants has undergone obvious expansion and contraction, which might be one of the reasons for the adaptation of plants of this genus to tropical-subtropical climates (Huang et al. 2023). The genomic studies of *Castanea* species have mainly concentrated on Chinese Chestnut (*Ca. mollissima*), a species of significant economic and ecological value. The first genome sequence of *Ca. mollissima* was released on the Hardwood Genomics website in 2014. Subsequently, the genomes of this genus, including *Ca. dentata* (Westbrook et al. 2020), *Ca. crenata* (Shirasawa et al. 2021; Wang et al. 2022), *Ca. sativa* (Bianco et al. 2024), and other version of *Ca. mollissima* (Xing et al. 2019; Staton et al. 2020; Sun et al. 2020; Wang et al. 2020), have been published. The limited availability of genomic data for *Castanopsis* and *Castanea* obscures our understanding of the evolutionary processes within these genera.

**Fig. 1:**
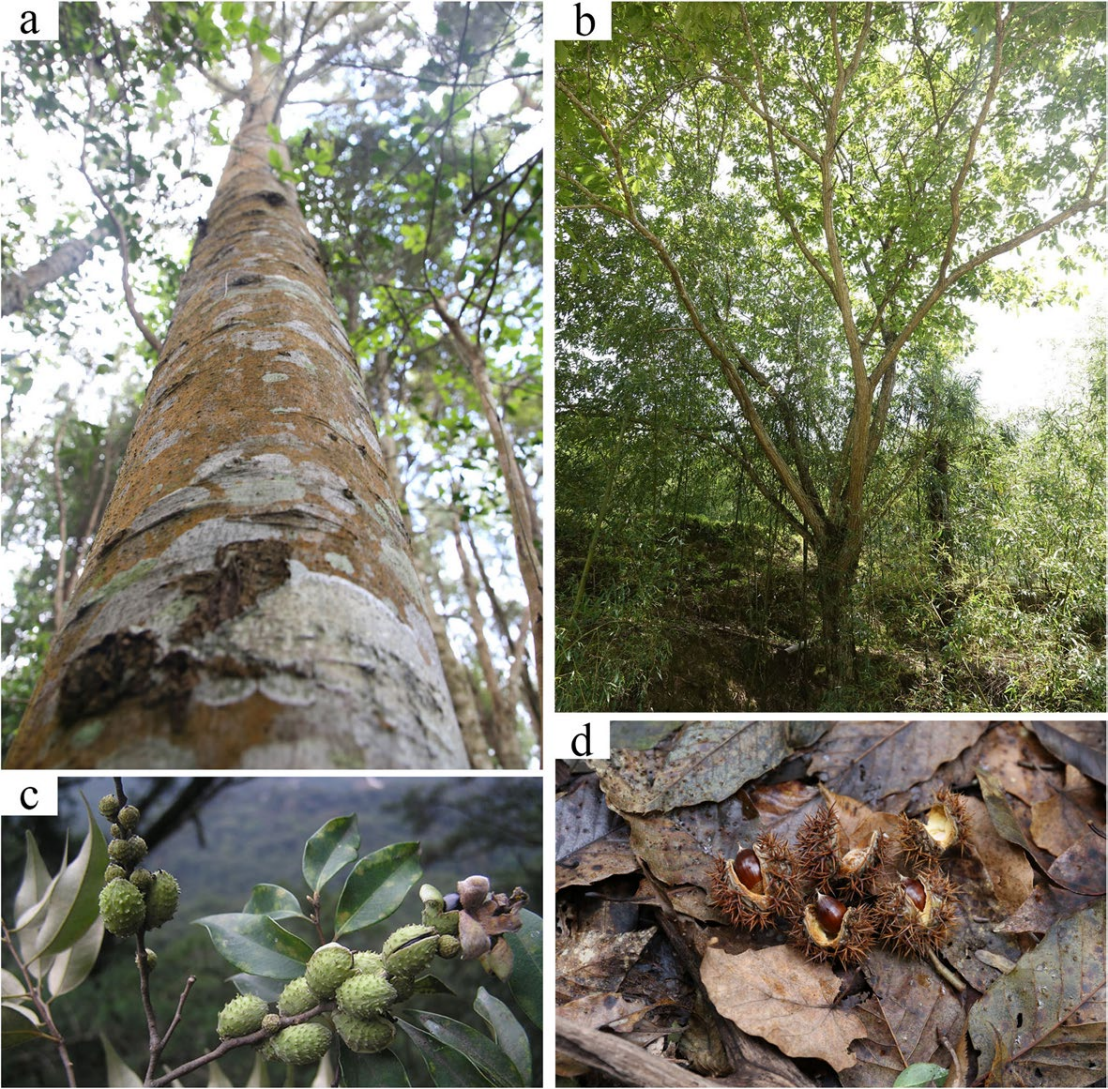
The morphological characteristics of *C. carlesii Ca. henryi. ***a** The overall morphological characteristics of *C. carlesii. ***b** The overall morphological characteristics of *Ca. henryi. ***c** The morphological characteristics of the fruit of *C. carlesii. ***d** The morphological characteristics of the fruit of *Ca. henryi*

*Castanopsis carlesii* (Hemsl.) Hay. (Fig. 1a and c) are widely distributed in the southern region of the Yangtze River in China. It is a dominant tree species in subtropical evergreen broad-leaved forests and has significant ecological value (Lin et al. 2017). Chinese chinquapin, *Castanea henryi* (Skam) Rehder & E.H. Wilson (Fig. 1b and d), is a dominant or subdominant tree species in the mountainous regions south of the Yangtze River in China (Lang et al. 2007), and its wood and nuts have substantial economic significance. Recently, we published the complete mitochondrial genome of these two species (Tu et al. 2024), and the plastid genomes reported by other researchers. The two species currently lack the largest nuclear genome in the three genomic sets of plants. In this study, we present the assembly of the chromosome-scale genomes of *C. carlesii* and *Ca. henryi*. These were complemented with published genomes to characterize the Fagaceae genome evolution. Our analyses of flower development, cupule formation, and starch-rich fruit development in *C. carlesii*, along with the identification of NLR genes within the Fagaceae family, enhance our understanding of the evolution of vital traits in Fagaceae. This study lays the foundation for a comprehensive understanding of the evolution and genetic resources of the family Fagaceae.

## Results

### Genome assembly and annotation

**Fig. 2:**
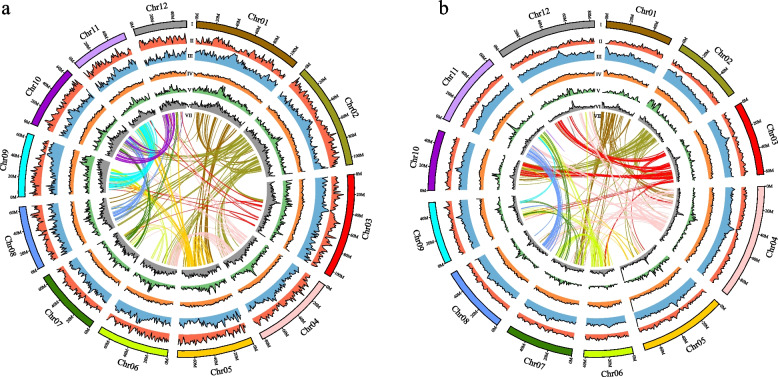
The features of the genome assembly of *C. carlesii* (**a**) and *Ca. henryi* (**b**). (I) chromosome, (II) gene density, (III) TE density, (IV) GC content, (V) Copia density, (VI) Gypsy density, (VII) collinearity

The genome of *C. carlesii* and *Ca. henryi* were sequenced and assembled using a combination of Illumina short reads, PacBio long reads, and Hi-C scaffolding (Table S1). Illumina sequencing generated 108.14 Gb and 63.11 Gb of paired-end reads for *C. carlesii* and *Ca. henryi*, respectively, providing estimates of genome size, repetitiveness, and heterozygosity. Survey analysis revealed genome sizes 899.76 Mb with 1.59% heterozygosity for *C. carlesii* and 755.49 Mb with 2.52% heterozygosity for *Ca. henryi* (Fig. S1). For complete genome sequencing, we generated 93.38 and 79.20 Gb of raw data for *C. carlesii* and *Ca. henryi*, respectively, using multiple insert libraries with PacBio technology (Table S1). The total genome assembly sizes were 927.24 Mb with a contig N50 value of 1.57 Mb for *C. carlesii* and 780.10 Mb with a contig N50 value of 1.07 Mb for *Ca. henryi* (Table S1). Collectively, 81.14 and 63.11 Gb of Hi-C sequencing data were generated for *C. carlesii* and *Ca. henryi*, respectively (Table S1). Based on assembled contigs, chromosome-scale scaffolding analysis grouped them into 12 pseudochromosomes with a total length of 912.16 Mb (99.76%) for *C. carlesii* (Fig. 2a) and 752 Mb (98.92%) for *Ca. henryi* (Fig. 2b). The lengths of the pseudochromosomes in *C. carlesii* and *Ca. henryi* ranged from 53.03 Mb to 107.51 Mb (N50 of 75.70 Mb) and 38.67 Mb to 95.10 Mb (N50 of 68.25 Mb), respectively (Table S1 and S2). The heat map of Hi-C interactions shows that the genome assemblies of *C. carlesii* and *Ca. henryi* were intact and robust (Fig. S2). A Benchmarking Universal Single-Copy Orthologs (BUSCO) assessment indicated that the *C. carlesii* and *Ca. henryi* genomes were 93.8% and 93.0% complete, respectively (Table S3). The average LTR Assembly Index (LAI) values for the whole genome of *C. carlesii* and *Ca. henryi* are 18.73 and 14.63, respectively (Fig. S3, Table S4).

We annotated 31,623 and 37,973 protein-coding genes in *C. carlesii* and *Ca. henryi* genome, respectively (Table S5). Based on BUSCO assessment, the protein-coding genes of *C. carlesii* were estimated to be 90.62% complete, and those of *Ca. henryi* were estimated to be 92.95% complete (Table S6). In total, 30,461 (96.33%) protein-coding genes were annotated in five functional databases to *C. carlesii* and 37,093 (97.68%) were annotated to *Ca. henryi* (Table S7). Furthermore, we identified 158 and 127 miRNAs, 635 and 777 transfer RNAs, 207 and 266 ribosomal RNAs, and 577 and 307 small nuclear RNAs in the respective genomes of *C. carlesii* and *Ca. henryi* (Table S8). There were 45.79% and 44.88% repetitive sequences in *C. carlesii* and *Ca. henryi* genomes, with long terminal repeats retrotransposons (LTR-RTs) accounting for 39.29% (16.35% Copia, 15.64% Gypsy, and 7.30% other) and 35.10% (12.19% Copia, 21.48% Gypsy, and 1.43% other) of the *C. carlesii* and *Ca. henryi* genomes (Table S9). To trace the insertion history of LTR-RTs in the Fagaceae family, we analyzed the LTR-RT insertion times of twelve species (Fig. S4). We found that in the genomes of *Castanopsis*, *Castanea*, and *Quercus*, LTR-RTs continued to accumulate in large quantities during the last 6 Mya, with a sub-peak appearing at approximately 3 Mya, and the number of effective insertions increased with time. In the genome of *Fagus sylvatica*, LTR-RTs gradually accumulated during the last 4 Mya, the effective insertion number increased rapidly with time, and its expansion period was relatively short compared to that of the other seven species.

### Gene family evolution

**Fig. 3:**
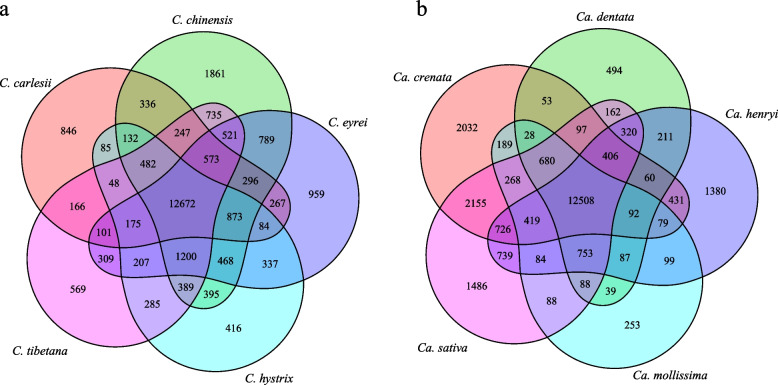
Venn diagram showing shared and unique gene families among five *Castanopsis* (**a**) and five *Castanea* (**b**) genomes

To investigate the evolutionary history of the *Castanopsis* and *Castanea* gene families, gene family clustering evaluation was performed on the whole genomes of five species from each genus. The five genomes of *Castanopsis* share 12,672 gene families, with 846 gene families being specific to *C. carlesii* (Fig. 3a). The five genomes of *Castanea* share 12,508 gene families, with 1380 gene families being specific to *Ca. henryi* (Fig. 3b).

**Fig. 4:**
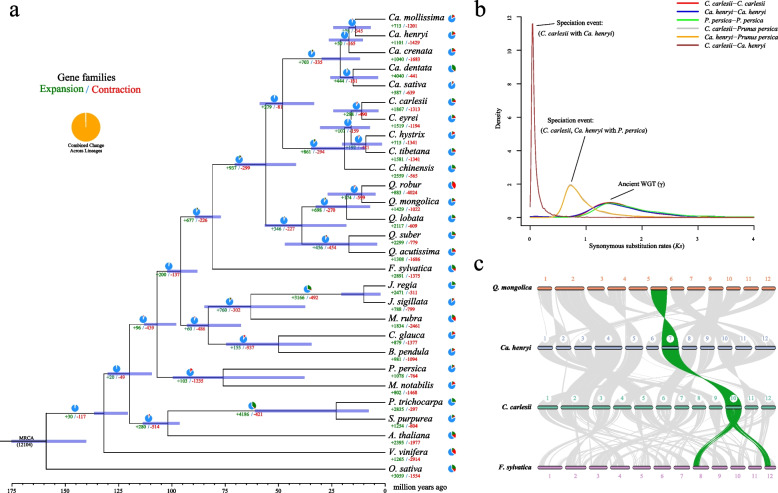
Comparative genomic analyses of *C. carlesii* and *Ca. henryi* with other plants. **a** Gene family evolution shown based on Bayesian tree. The quantities of expanded and contracted gene families are represented by green and red numbers, respectively. In the pie chart, the blue sections indicate gene families with consistent copy numbers, and while the orange portion signifies the 12,104 gene families identified in the most recent common ancestor (MRCA). **b** *Ks* distribution in *C. carlesii*, *Ca. henryi* and *P. persica.* The peak in the intraspecific *Ks* distribution signifies ancient whole-genome duplication events, and the peak in the interspecific *Ks* distribution denotes species divergence events. **c** Syntenic analysis of the 12 chromosomes of *C. carlesii*, *Ca. henryi*, *F. sylvatica* and *Q. mongolica*. The grey lines connecting chromosomes represent syntenic links, and collinear relationship is emphasized by a syntenic set displayed in green

We constructed a phylogenetic tree and estimated the divergence times of 28 species, including 16 species from Fagaceae (Fig. 4a and Table S10). Phylogenetic analysis revealed that the species within the Fagaceae family formed a distinct clade, with *F. sylvatica* at its base. The Juglandaceae + Myricaceae and Betulaceae + Casuarinaceae clades initially formed sister groups and later emerged as sister groups to Fagaceae. Notably, *C. carlesii* was shown to be sister to *C. eyrei* and *Ca. henryi* was shown to be sister to *Ca. mollissima*. The estimated divergence time between *Castanopsis* and *Castanea* was determined to be 48.3 (33.35–58.78) Mya (Fig. 4a).

Comparison of species in the Fagaceae revealed that gene families expanded more significantly than they contracted before the large-scale species divergence (Fig. 4a). In the Fagaceae family, 677 gene families underwent expansion, whereas 226 gene families underwent contraction. Within the *Quercus*-(*Castanea*-*Castanopsis*) clade, 937 and 299 gene families expanded and contracted, respectively. In the *Castanopsis* clade, 861 and 294 gene families underwent expansion and contraction, respectively. In the *Castanea* clade, 703 and 335 gene families underwent expansion and contraction, respectively.

Gene ontology (GO) enrichment analysis revealed the unique gene families of Fagaceae to be specifically enriched in terms “ADP binding”, “defense response”, “signal transduction” and “response to stimulus” and the Kyoto Encyclopedia of Genes and Genomes (KEGG) pathway was enriched for the genes involved in “Fanconi anemia pathway” and “galactose metabolism” (Table S11). GO and KEGG enrichment analyses found that the significantly expanded gene families of *Castanopsis* were particularly enriched in “defense response” and “plant-pathogen interaction” (Table S12). In *Castanea*, GO and KEGG enrichment analyses are particularly enriched in “heme binding”, “mismatch repair” and “DNA replication” (Table S12).

### Whole-genome duplication and collinearity

The distribution of synonymous substitutions per synonymous site (*Ks*) for paralogous genes in *C. carlesii* and *Ca. henryi* shows a prominent peak at *Ks* 1.4–1.5 (Fig. 4b), similar to the *Ks* value observed in *Prunus persica*. Furthermore, the *Ks* differentiation peaks between *C. carlesii* and *P. persica*, *Ca. henryi* and *P. persica*, and *C. carlesii* and *Ca. henryi* were smaller than the *Ks* peaks in *P. persica*, *C. carlesii*, and *Ca. henryi* (Fig. 4b). This suggests that the common ancestors of *P. persica*, *C. carlesii*, and *Ca. henryi* experienced a polyploidy event before the divergence of *C. carlesii*, *Ca. henryi*, and *P. persica*. Therefore, we conclude that the ancestors of *C. carlesii* and *Ca. henryi* experienced only the γ event, and *C. carlesii* and *Ca. henryi* did not undergo separate polyploidy events.

We examined the collinearity between *C. carlesii*, *Ca. henryi*, *Q. mongolica*, and *F. sylvatica*. The results demonstrate that *Ca. henryi* exhibited a high collinearity with *C. carlesii* and *Q. mongolica* (Fig. 4c and Table S13), suggesting the absence of significant large-scale structural variations after the divergence of *Castanopsis*, *Castanea*, and *Quercus*. However, each chromosome of *C. carlesii* was collinear with multiple chromosomes of *F. sylvatica* (Fig. 4c and Table S13), indicating a more distant relationship between *Fagus* and *Castanopsis*, *Castanea*, and *Quercus*.

### Flower development

**Fig. 5:**
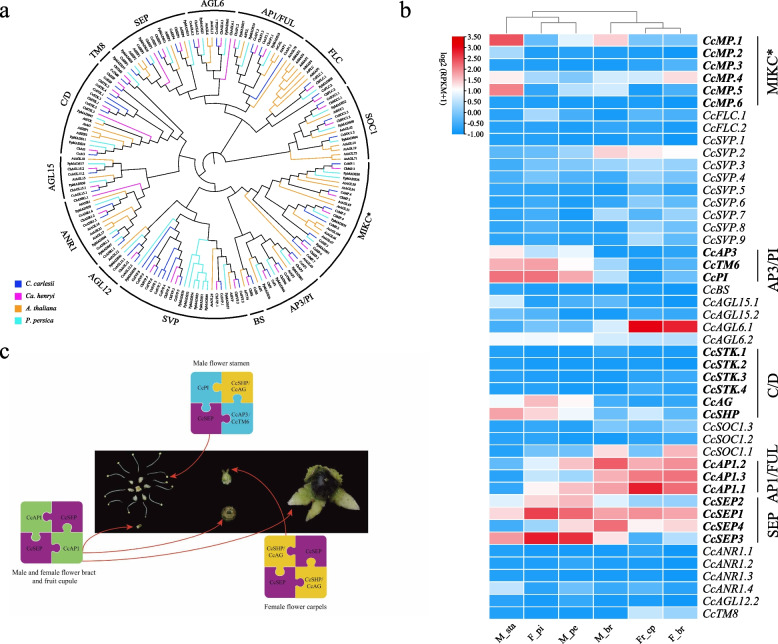
Development of *C. carlesii* flower. **a** Phylogenetic analysis of MADS-box Type II genes from *C. carlesii*, *Ca. henryi*, *A. thaliana*, and *P. persica*. **b** The expression patterns of MADS-box Type II genes in reproductive and vegetative organs of *C. carlesii*. M_pe: male flower perianth; M_br: male flower bract; M_sta: male flower stamen; F_pi: female flower stylopodium; F_br: female flower bract; Fr_cp: fruit cupula. **c** Flower model of *C. carlesii*

A total of 69 and 57 MADS-box genes were identified in the genome of *C. carlesii* and *Ca. henryi*, respectively (Table S14). Phylogenetic analysis grouped these genes into two types: types I and types II (Fig. S5). Within type II, the genes were further divided into MIKC* and MIKCc (Fig. 5a). Compared to *Arabidopsis thaliana* (106) and *P. persica* (78), the number of MADS-box genes in Fagaceae species was lower (Table 1). The number of type II genes in Fagaceae ranged from 29 (*F. sylvatica*) to 49 (*C. carlesii*), and this gap was due to the number of *AGAMOUS* (*AG*) and *SHORT VEGETATIVE PHASE* (*SVP*). The AG subfamily was divided into the *AG* and *AGAMOUS-LIKE11* (*AGL11*) lineages (Fig. S6a). In the *AGL11* lineage, the Fagaceae expanded, and *C. carlesii* and *Ca. henryi* had four and three members, respectively. The *SVP* were divided into three clades: SVP1, SVP2, SVP3, and in Fagaceae, the MADS-box gene has one member each in the SVP1 and SVP2 branches, but it expands into the SVP3 clade (Fig. S6b). However, not all species within the Fagaceae family show an expansion of *SVP* genes in SVP3. We observed this expansion in *Castanopsis* and *Castanea*, as well as in some species within *Quercus*. The gene extensions on SVP3 clade in *C. carlesii* and *Ca. henryi* were found to be arranged in tandem on chromosomes 8 and 1, respectively (Fig. S7).

**Table 1:** MADS-box genes in *A. thaliana*, *P. persica*, *C. carlesii*, *C. tibetana*, *Ca. henryi*, *Ca. mollissima*, *Q. acutissima*, *Q. mongolica*, and *F. sylvatica*

Category	*A. thalianaa*	*P. persica*	*C. carlesii*	*C. tibetana*	*Ca. henryi*	*Ca. mollissima*	*Q. acutissima*	*Q. mongolica*	*F. sylvatica*
Total	106	78	69	65	58	61	58	69	54
Type I (Total)	60	40	20	31	21	23	21	23	25
Mα	24	21	11	19	13	15	14	13	17
Mβ	20	7	5	7	4	5	1	7	3
Mγ	16	12	4	5	4	3	6	4	5
Type II (Total)	46	38	49	34	37	38	37	46	29
MIKC*	7	4	7	5	4	4	6	7	4
MIKCc	39	34	42	29	33	34	31	39	25
*AP1/FUL*	4	3	3	2	3	3	4	3	3
*AP3/PI*	2	3	3	3	3	3	3	3	3
*Bs*	2	0	1	1	1	1	1	0	1
*AG-C/D*	4	3	6	4	5	5	4	7	3
*SEP*	4	4	4	3	3	4	4	4	3
*FLC*	6	1	2	1	2	2	2	2	2
*SVP*	2	8	9	8	5	7	2	5	2
*AGL15*	2	2	2	1	2	2	1	2	2
*AGL6*	2	2	2	1	3	2	1	2	2
*AGL12*	1	1	2	1	1	1	1	1	0
*TM8*	0	1	1	1	1	1	1	1	1
*ANR1*	4	3	4	2	2	2	4	4	1
*SOC1*	5	3	3	1	2	1	3	4	2

To gain insight into the flower development in *Castanopsis*, we conducted transcriptome sequencing of male and female mature flower components (stamens, carpels, petals, and sepals) and cupules of *C. carlesii*. The transcriptome sequencing of floral components yielded an average of 6.25 clean Gb, with a Clean Reads Ratio of 99.28%. It is worth noting that we found the existence of degenerate stamens similar to those in the female flowers of *Castanea* in the female flowers of *C. carlesii* (Fig. S8). The A-class gene, *CcAP1*, is expressed in male perianth, male bracts, female column base, and fruit cupule of *C. carlesii* (Fig. 5b). B-class genes, including *CcAP3*, *CcPI*, and *CcTM6*, were significantly expressed in the male stamens, female column bases, and male petals of *C. carlesii* (Fig. 5b). It is notable that B-class gene *CcPI* are also expressed at the initial stage of female flowers (Fig. 5b). The C/D-class genes in *C. carlesii* include *CcSHP*, *CcAG*, and *CcSTK.1/2/3/4*. Among these, *CcSTK.1/2/3/4* showed no expression across the various stages of male and female inflorescences and flower structures (Fig. 5b). E-class genes *CcSEP1* and *CcSEP3* were highly expressed in the stamens, perianth of male flowers, and female flower stylopodium, whereas *CcSEP1* and *CcSEP4* were expressed in the bracts of male and female flowers (Fig. 5b).

### Fruit development

Sucrose-starch metabolism is regulated by a series of enzymes, including genes related to sucrose metabolism, starch synthesis, and starch degradation. Based on the morphological anatomy, we divided the fruit development of *C. carlesii* into seven stages (Fr1–Fr7) (Fig. S9). Select four periods (Fr1, Fr3, Fr5, and Fr7) for transcriptome sequencing, with three replicates for each period. Finally, the transcriptome sequencing at four fruit stages yielded an average of 6.44 clean Gb, with clean reads ratio of 94.59%. To explore the relationship between starch accumulation and sucrose content, we analyzed the dynamic changes in sucrose and starch content during *C. carlesii* fruit development in seven periods, and there was a clear negative correlation between sucrose and starch content changes (Fig. S10).

**Fig. 6:**
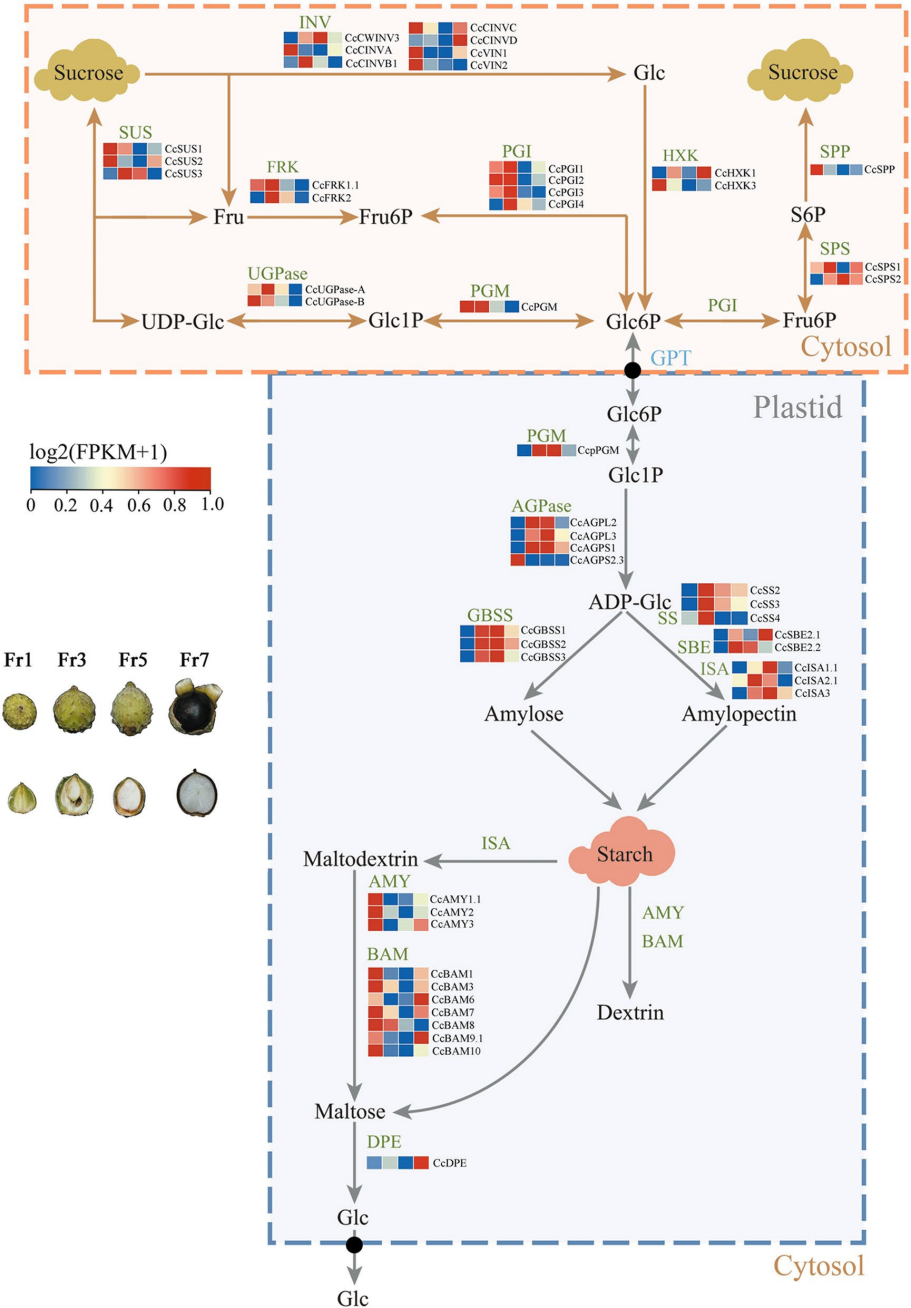
Gene expression profile associated with sucrose-starch metabolism in *C. carlesii*. Fr1, fruit development stage with the cotyledons are light green and transparent, occupying a small area of the entire nut. Fr3, fruit development stage with the cotyledon becomes larger, accounting for half of the nut. Fr5, fruit development stage with the cotyledon continues to grow and fill the whole nut. Fr7, fruit development stage with the skin turns black and the fruit ripens

Nine types of 36 sucrose metabolism-related enzyme genes were identified in the genome of *C. carlesii* (Table S15). The sucrose content of *C. carlesii* fruits decreased sharply during Fr2–Fr3 stages (Fig. S10, Table S16), and the genes related to sucrose degradation enzymes, including *CcCINVB1*, *CcSUS3*, *CcFRK1.1/2*, *CcPGI1/2/3/4*, *CcUGPase-A*, *CccPGM* and *CcHXK3*, were mainly expressed at the Fr3 stages (Fig. 6), indicating that these genes may be involved in the sucrose degradation process in *C. carlesii* fruits. The sucrose content in *C. carlesii* fruits increased during Fr3–Fr7 stages (Fig. S10), and *CcSPS2* was highly expressed during Fr3–Fr7 (Fig. 6), suggesting that *CcSPS2* may be related to sucrose synthesis in *C. carlesii* fruits.

Five types of 25 enzyme genes related to starch synthesis were identified in the genome of *C. carlesii* (Table S15). Most starch synthase-related genes, including *CcpPGM*, *CcAGPL2/3*, *CcAGPS1*, *CcGBSS1/2/3*, *CcSS2/3/4*, *CcSBE2.2*, and *CcISA2.1*, were sharply upregulated during Fr3, followed by a sustained high expression during Fr5 and subsequent downregulation during Fr7 (Fig. 6). This was consistent with the trend of the measured starch content (Fig. S10), indicating that the expression of these genes is closely related to starch synthesis in *C. carlesii* fruits.

Seventeen starch degradation-related enzyme genes were identified in the genome of *C. carlesii* (Table S15). The identified differentially expressed genes *CcAMY1.1/2/3* and *CcBAM1/3/7/8/10* were highly expressed during Fr1 (Fig. 6). Conversely, the expressions of *CcAMY3*, *CcBAM6/9.1*, and *CcDPE*, which regulate starch degradation, was upregulated during Fr7 (Fig. 6). Concurrently, the starch content decreased during the Fr5–Fr7 stages (Fig. S10), suggesting that *CcAMY3*, *CcBAM6/9.1*, and *CcDPE* were closely associated with starch degradation towards the end of fruit development.

### Evolution of NLR-type resistance genes

**Fig. 7:**
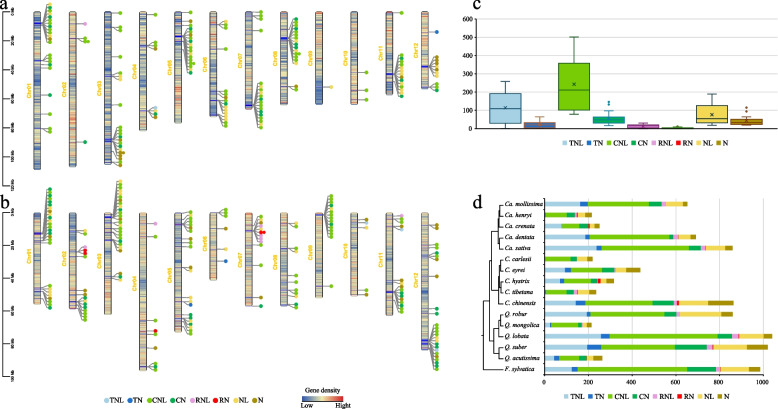
Nucleotide-binding leucine-rich repeat (NLR) genes identified in Fagaceae. **a** NLR genes located in the chromosomes of *C. carlesii*. **b** NLR genes located in the chromosomes of *Ca. henryi*. **c** Boxplot of the quantity of 8 types of NLR genes in each species of the Fagaceae family. **d** Comparison of the quantities of NLR genes in the Fagaceae, with the phylogenetic relationships on the left

**Fig. 8:**
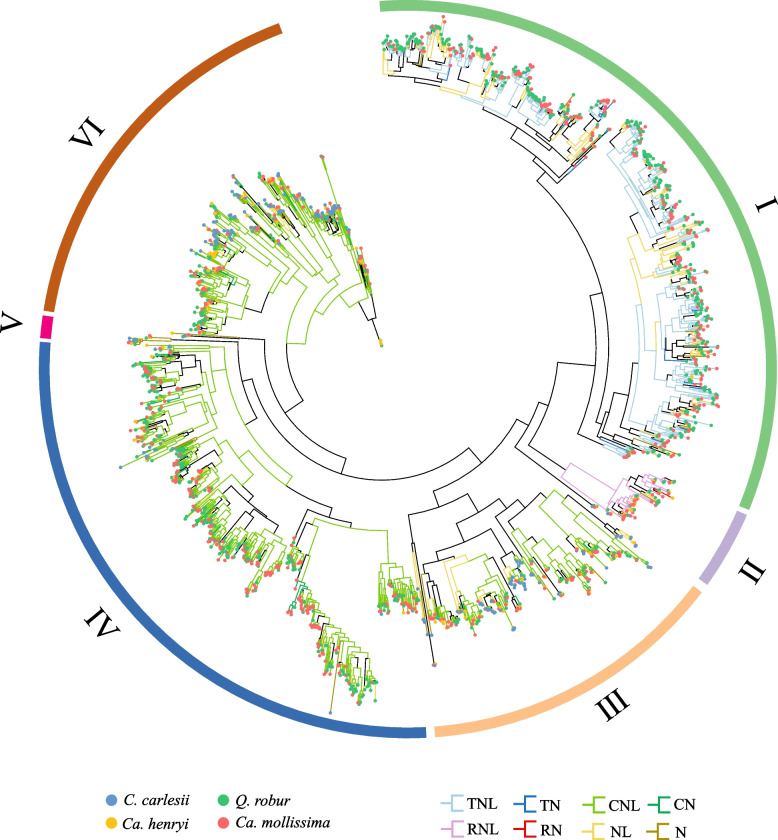
Phylogenetic analysis of Nucleotide-binding leucine-rich repeat (NLR) genes in four species of Fagaceae based on NB-ARC domain. Show the legend below, with species represented by four different circles and branch colors representing different types of NLR genes

Multiple disease resistance-related genes are induced in response to stress and increase Fagaceae tolerance, making some members of this family well-suited for addressing fundamental questions on the nature of host–pathogen genome co-evolution and the biology of invasive pathogens. In this study, we identified and classified NLR genes in 12 representative Fagaceae plants to investigate the characteristics and evolutionary patterns of NLR genes in Fagaceae species (Table S17). These NLR genes are classified into eight types: TNL, TN, CNL, CN, RNL, RN, NL, and N. The NLR gene families in *C. carlesii* and *Ca. henryi* were unevenly distributed across chromosomes, with pronounced clustering in specific regions. the distribution of these NLRs on chromosomes is uneven and mostly clustered (Fig. 7a and b). Comparative analysis revealed 215–1040 NLR genes across 12 Fagaceae species, with significant expansions in CNL, TNL and NL types (Fig. 7c). There are significant differences in NLRs among species of Fagaceae plants, and NLRs species with significant expansion do not cluster together (Fig. 7d). To investigate the factors contributing to differences in NLRs, we constructed a phylogenetic tree based on the Nucleotide binding domain (NB-ARC) in the NLRs from four species: *C. carlesii* and *Ca. henryi*, which have low NLR numbers, as well as *Q. robur* and *Ca. mollissima*, which have high NLR numbers. For clarity, we divided the phylogenetic tree into six branches (Fig. 8), with branches I and IV being the largest. In branch I, the main types of NLRs include NL and TNL, with *Q. robur* and *Ca. mollissima* being the predominant species with high numbers of NLRs. In branch IV, the main type of NLR is CNL. Similar to branch I, *Q. robur* and *Ca. mollissima* are the predominant species with high NLRs counts. There were no other branches observed that showed such significant differences in species composition as seen in branches I and IV.

## Discussion

In the Fagaceae family, *Castanopsis* and *Castanea* are two closely related genera, with great ecological and economic value. Despite their importance, genomic resources for these genera remain limited compared to the extensively studied *Quercus*. To bridge this gap, we employed Illumina and PacBio reads with Hi-C technology to obtain the whole genomes of *C. carlesii* and *Ca. henryi*. The final assembled genome size of *C. carlesii* is 927.24 Mb, with a BUSCO completeness of 93.8%. Hi-C scaffolding grouped 99.76% of the *C. carlesii* genome into 12 pseudochromosomes, ranging from 53.03 Mb to 107.51 Mb. The *C. carlesii* genome contains a total of 30,461 protein-coding genes, 158 miRNAs, 635 tRNAs, 207 rRNAs, and 577 snRNAs. The final assembled genome size of *Ca. henryi* is 780.10 Mb, with a BUSCO completeness of 93.0%. Hi-C scaffolding grouped 98.92% of the *Ca. henryi* genome into 12 pseudochromosomes, ranging from 38.67 Mb to 95.10 Mb. The *Ca. henryi* genome contains a total of 37,093 protein-coding genes, 127 miRNAs, 777 tRNAs, 266 rRNAs, and 307 snRNAs. Significant differences exist in the published BUSCO values between the genomes of *Castanopsis* and *Castanea*, with *C. tibetana* at 92.95% (Sun et al. 2022), *Ca. mollissima* at 92.44% (Wang et al. 2020), and *C. hystrix* at 99.50% (Huang et al. 2023). We further evaluated the genome assembly quality using LAI values. The LAI values for the whole genomes of *C. carlesii* and *Ca. henryi* were 18.73 and 14.63, respectively, indicating that the assembly quality reached reference standards. Transposable elements (TEs) constitute a significant portion of plant genomes and play a crucial role in evolution (Bennetzen and Wang 2014). The genomes of *C. carlesii* and *Ca. henryi* contain similar proportions of repetitive sequences, 45.79% and 44.88%, respectively. The proportion of repetitive elements varies significantly across Fagaceae genomes. For example, *C. chinensis* (55.9%) (Chen et al. 2023), *C. hystrix* (50.95%) (Huang et al. 2023), *Ca. mollissima* (44.16%) (Sun et al. 2020), *Ca. mollissima* (44.90%) (Qiao et al. 2024), *Ca. crenata* (59.00%) (Shirasawa et al. 2021), *Quercus suber* (35.89%) (Ramos et al. 2018), and *F. sylvatica* (60.36%) (Mishra et al. 2018) all show considerable variation in repetitive sequence proportions. It is important to note that the quality of genome assembly and the methods used to identify repetitive sequences may influence their proportion. LTR-RTs insertion time analysis showed that the plants of the Fagaceae family experienced a large-scale LTR-RTs outbreak, but there were differences in the time and degree of LTR-RTs expansion, especially *F. sylvatica* compared to other species.

The expanded gene family in *Castanopsis* is predominantly associated with stress resistance, potentially contributing to its ecological success and diversity in subtropical and tropical forests. These expanding gene families may provide important clues regarding the evolution of the Fagaceae genome. Phylogenetic analysis based on single-copy homologous genes reveals that the clades of Juglandaceae + Myricaceae and Betulaceae + Casuarinaceae initially formed sister groups and subsequently emerged as sister groups to Fagaceae, which is consistent with the APG IV classification (APG IV 2016). Within this phylogenetic framework, relationships at the genus level further illustrate specific affiliations: *C. carlesii* is most closely related to *C. eyrei*, while *Ca. henryi* shows the strongest association with *Ca. mollissima*. Notably, the divergence between *Castanopsis* and *Castanea*, estimated at approximately 48.3 Mya. We found that *C. carlesii* and *Ca. henryi* did not undergo Whole genome duplication (WGD) after the triploid reproductive event, a feature shared by most eudicots (Cheng et al. 2018). Based on comparative genome analysis, we found that the genomes of *C. carlesii*, *Q. mongolica*, and *Ca. henryi* are highly homologous compared to *F. sylvatica*.

The MADS-box family is widely recognized for its pivotal roles in various crucial processes of plant development, particularly flowering and flower development (Causier et al. 2002; Bai et al. 2019). The number of type II genes in Fagaceae ranged from 29 (*F. sylvatica*) to 49 (*C. carlesii*), and this gap was main due to the number of *AG* and *SVP*. The AG subfamily was divided into the *AG* and *AGL11* lineages, which belong to the MADS-box C/D-class, are pivotal in regulation of floral organs, floral meristems, and fruit development. In the *AGL11* lineage, the Fagaceae expanded, and *C. carlesii* and *Ca. henryi* had four and three members, respectively. In *Arabidopsis*, four AG subfamily genes have been identified, among which *SEEDSTICK* (*STK*) is the only member of the *AGL11* lineage and is specifically expressed in the ovule (Pinyopich et al. 2003). The expansion of Fagaceae in *AGL11* lineage might have contributed to the development of Fagaceae fruit ovules. The *SVP* were divided into SVP1, SVP2, and SVP3 clades. In *Arabidopsis*, there are two *SVP* paralogs, *SVP* and *AGAMOUS-LIKE24* (*AGL24*). *SVP* inhibits the flowering process and *AGL24* acts as a flowering activator (Hartmann et al. 2000; Yu et al. 2002; Liu et al. 2018). In *P. persica*, the *Dormancy-associated MADS-box* (*DAM*) gene has six extensions in the SVP2 clade, and deletion mutations of these six *SVP* homologs result in the complete absence of dormancy (Bielenberg et al. 2008). In Fagaceae, the MADS-box gene expands into the SVP3 clade, such as *Populus trichocarpa* and *Glycine max*, rather than in the absence of SVP3 in Fagaceae (Liu et al. 2018). However, not all species within the Fagaceae family show an expansion of *SVP* genes in SVP3. We observed this expansion in *Castanopsis* and *Castanea*, as well as in some species within *Quercus*. The SVP3 group was highly expressed in vegetative organs such as buds, leaves, cotyledons, and dormant buds of grapes, poplars, and apples, indicating that the SVP3 group is involved in the dormancy process (Liu et al. 2018). Amplification of SVP3 group genes in Fagaceae may be involved in their dormancy.

Following the ABCDE model of flower development, the specification of the four floral organ types in a typical flower involved five classes of floral organ identity genes: A + E for sepals, A + B + E for petals, B + C + E for stamens, C + E for carpels, and C + D + E for ovules (Theißen et al. 2016). The expression patterns of B-class genes *CcAP3* and *CcTM6* in *C. carlesii* resembled those in *Ca. sativa* and *Q. suber*: *AP3* and *TM6* exhibited high expression in male flowers and low expression in female flowers (Sobral and Costa 2017; Alhinho et al. 2021). However, *PI* was expressed in female flowers of both *C. carlesii* and *Ca. sativa* (Alhinho et al. 2021). Notably, this expression pattern was not observed in *Q. suber*, which is potentially attributable to the presence of staminodes in female flowers of *C. carlesii* and *Ca. sativa*. In *Ca. sativa* and *Q. suber*, PI exhibited the ability to interact with AP3 and TM6; this interaction is thought to be conserved in Fagaceae. The C/D-class genes *CcAG* and *CcSHP* displayed similar temporal expression patterns; however, the expression of *CcSHP* was notably higher than that of *CcAG*. In *Ca. sativa* and *Q. suber*, *SHP* was expressed during the late male flowering stage. *AG* and *SHP* shared similar temporal expression patterns, and no interactions were observed between B- and AG-like proteins. This suggests that SHP may function as a C-like gene, requiring simultaneous action with AG to fulfill the role of C in stamen development (Sobral and Costa 2017; Alhinho et al. 2021). By considering the gene expression patterns of homologs associated with the ABCDE model of floral organ and cupule identities in *C. carlesii*, along with insights from previous studies, we deduced a model for floral and cupule development in *C. carlesii* (Fig. 5c): CcPI + CcSEP + CcAP3/CcTM6 + CcSHP/CcAG for male stamens, CcSHP/CcAG + SEP for female flower carpels, and CcAP1 + CcSEP for bracts and fruit cupules.

The main glucosyl donor for starch biosynthesis in heterotrophic sink organs is sucrose produced in leaves. This sucrose is transported from leaf tissue to developing heterotrophic sink tissue via the phloem. The pathway for sucrose metabolism requires the cleavage of sucrose by either sucrose synthase (SUS) or invertase (INV) (Mitsui et al. 2010). SUS typically catalyzes the reversible conversion of sucrose and uridine diphosphate (UDP) to produce fructose (Fru) and UDP-glucose (UDPG) in the cytoplasm of storage organs, while INV catalyzes the irreversible hydrolysis of sucrose to produce glucose (Glu) and Fru (Geigenberger and Stitt 1993). The high expression of *CcSUS3* during stages Fr3 is consistent with the rapid decrease of sucrose during the development of *C. carlesii* fruit, indicating that *CcSUS3* is the main enzyme responsible for initial sucrose cleavage during this fruit development phase. The high expression levels of *CcFRK1.1/2* and *CcUGPase-A* at this stage suggest that there is strong transcriptional coordination between *CcSUS3*, *CcFRK1.1/2*, and *CcUGPase-A*, which may contribute to the production of the cytosolic hexose phosphate pool (glucose-1-phosphate [G1P] and fructose-6-phosphate [F6P]) during *C. carlesii* fruit development, consistent with findings from studies on *Ca. mollissima* (Shi et al. 2021). In addition to SUS, we observed abundant INV transcripts during stages Fr1–Fr3, and similar trends in sucrose degradation during fruit growth, indicating that these enzymes may also contribute to sucrose metabolism in *C. carlesii* fruit, as observed in *Ca. mollissima* fruits (Shi et al. 2021). The co-regulation of SUS and INV activities may enable specific responses to carbon allocation, thereby driving the growth, development, and starch accumulation in developing *C. carlesii* fruit.

Numerous members of this family face a range of environmental stresses, notably from fungal and fungal-like diseases, such as *Phytophthora* spp. and *Cryphonectria parasitica* (Staton et al. 2015). Plants possess a wealth of resistance (R) genes that protect them from various pathogens. The largest R gene family consists of genes encoding receptors with NB-ARC domain, which are known as NLR or NBS-LRR genes (Eitas and Dangl 2010; Lee and Yeom 2015). The analysis of the distribution of NLR genes shows that they mostly cluster on chromosomes and are clustered in a small area of the chromosome. Previous studies, such as those in *Arabidopsis*, showed that most NLR genes on chromosomes occur in clusters (Meyers et al. 2003). The formation of NLR gene clusters is believed to provide synergistic regulatory benefits (van Wersch and Li 2019). NLR gene amplification was observed across multiple Fagaceae species, yet these expansions were not confined to a single phylogenetic clade, suggesting independent adaptation events to diverse pathogen pressures. Research suggests that in the absence of pathogen recognition, many plants may reduce the cost of resistance by eliminating resistance (Bergelson et al. 2001; Tian et al. 2003). There are studies indicating that the significant contraction of the R gene family in *Quercus* may be related to the loss of certain pathogenic bacteria in East Asian environments (Ai et al. 2022). We constructed a phylogenetic tree of NLRs from four representative Fagaceae species with significant differences in NLRs quantity, and found that the two largest branches, I and II, were predominantly composed of *Q. robur* and *Ca. mollissima*, which exhibited significantly higher numbers of CNL, TNL, and NL types of NLRs. It is the significant expansion of these three types of NLRs that leads to the difference in NLRs quantity among Fagaceae species. We believe that the expansion of similar NLRs gene types in Fagaceae species is an adaptation to similar environmental pressures.

In conclusion, we have assembled chromosome‑level genomes of *C. carlesii* and *Ca. henryi* using Illumina, PacBio, and Hi-C technologies. Comparing and analyzing the genomes of other species in the Fagaceae family provides further insight into the evolution of the family. Transcriptome sequencing of the flowers, cupules, and fruits of *C. carlesii* is crucial for understanding the expression patterns of MADS-box genes in flower development, cupule formation, and sucrose-starch related genes in fruit development within this family. Additionally, our findings suggest that NLR gene expansion is similar across species, likely as an adaptation to similar environmental pressures. However, the study has limitations, particularly the lack of functional validation for some genes. Future research will focus on conducting experiments on specific genes in these areas to confirm their functions.

## Material and methods

### Sample preparation and sequencing

Plant materials for genome and transcriptome sequencing were collected from wild *C. carlesii* in Gushan Mountain, Fuzhou City, Fujian Province, China (26° 05′ N, 119° 39′ E) and wild *Ca. henryi* in Nanping City, Fujian Province, China (27° 02′ N, 118° 44′ E).

For genome sequencing, a modified cetyltrimethylammonium bromide method was used to extract total genomic DNA from young leaves. A 500 bp paired-end library was constructed following the Illumina protocol and sequenced on an Illumina HiSeq 2000 platform. Additionally, we performed 20-kb single-molecule real-time (SMRT) DNA sequencing using PacBio to sequence a DNA library on the PacBio Sequel platform. For Hi-C sequencing, a Hi-C library was prepared by Santa Cruz Biotechnology (Dovetail Genomics, Scotts Valley, CA, USA) and sequenced on an Illumina NovaSeq.

For transcriptome sequencing, RNA was extracted from fruits at four developmental stages, as well as from mature floral components (stamens, carpels, petals, and sepals) and cupules of *C. carlesii*. Transcriptome libraries were constructed using the Illumina TruSeq Library Stranded mRNA Prep Kit and sequenced on a Illumina HiSeq 2000 platform.

### Genome assembly

We evaluated the genome size of *C. carlesii* and *Ca. henryi* based on *K*-mer analysis to determine the amount of sequencing data required for genome assembly. GenomeScope (Vurture et al. 2017) was used to determine genome size and heterozygosity based on *K*-mer distribution. Based on the PacBio sequencing data after quality control, we used FALCON v1.8.7 assembler (https://github.com/PacificBiosciences/FALCON/) and Falcon-unzip (Chin et al. 2016) for genome assembly and phase haplotypes. FALCON first compares the reads with each other for error correction to generate a consensus sequence, then uses error-corrected reads to compare with each other again, finds the overlap between the reads to construct a map, and then generates a map-based assembled sequence. Thereafter, FALCON-Unzip was used to phase the haplotypes and generate consensus sequences for primary contigs and associated haplotigs. The draft assembly was polished using GenomicConsensus package (https://github.com/PacificBiosciences/GenomicConsensus) that contains a main driver program (variantCaller), provides two consensus/variant calling algorithms: Arrow and Quiver. BWA-MEM (Li 2013) was then used to map short-reads onto the genome assembly and base errors were corrected using Pilon v1.22 (Walker et al. 2014). However, FALCON-Unzip may not have completely typed the genome sequence, resulting in redundancy in the assembly results. Purge_haplotigs (Roach et al. 2018) removes redundancy for high heterozygous regions where two haplotypes are assembled into primary contigs separately based on the coverage of reads, and retains only one haplotype. The integrity of the assembled genome was evaluated using BUSCO v3 (Simão et al. 2015). For Hi-C data, we used SOAPnuke v1.5.3 (Chen et al. 2018) to filter the raw data obtained by Hi-C sequencing and obtain high-quality clean reads. Along with the Hi-C data, Juicer software (Durand et al. 2016) and 3D-DNA software (Dudchenko et al. 2017) were used to assemble and generate chromosome-level genomes. Finally, approximately 99.76% and 98.92% of sequences were grouped into 12 super scaffolds for *C. carlesii* and *Ca. henryi*, respectively. The LTR assembly index score was calculated by LAI tool wrapped in LTR_retriever v2.9.0 (Ou et al. 2018), and plotted using ggplot2 package (https://ggplot2.tidyverse.org) in R (v4.4.1).

### Identification of repetitive elements

Based on the homology prediction method, TEs were first identified using RepeatMasker v3.3.0 (http://www.repeatmasker.org) and RepeatProteinMask within Repbase v21.12 (Jurka et al. 2005). In addition, a de novo prediction method based on self-sequence alignment was developed using two prediction software programs, RepeatModeller (Price et al. 2005), RepeatScount, and was based on repeat sequence features using two prediction software programs, Tandem Repeats Finder v4.09 (Benson 1999) and LTR-FINDER v1.06 (Xu and Wang 2007). Finally, repetitive sequences with 50% identities were grouped into the same class. LTRharvest v1.5.10 (Ellinghaus et al. 2008) was used to identify the LTR-RTs in the genome, which were then classified using the DANTE tool on the Galaxy server (Neumann et al. 2019). The two ends of these LTR-RTs were aligned using MAFFT v7.490 (Katoh and Standley 2013) and the nucleotide distance (*K*) between them was determined using a distmat in the EMBOSS package (http://emboss.sourceforge.net/). Finally, the insertion time of LTR-RTs was calculated based on the following formula: *T* = (*K*/100)/2*r*)/1000000 (substitution rates (*r*) = 1.3E − 8 per site and per year) (Ma and Bennetzen 2004).

### Gene predictions and annotation

Prediction methods based on homology- and de novo-based predictions and transcriptome data have been used to predict protein-coding genes. The homologous proteins from six known whole genome sequences of *Q. suber*, *Q. robur*, *A. thaliana*, *E. grandis*, *V. vinifera*, and *A. trichopoda* were downloaded from Phytozome 13 (https://phytozome.jgi.doe.gov/pz/portal.html). These homologous proteins were aligned with those in *C. carlesii* and *Ca. henryi* genome sequence using GEMOMA v1.3.1 (Keilwagen et al. 2016). In addition, we used Augustus v3.1 (Stanke et al. 2006) and SNAP (version 2006–07–28) (Johnson et al. 2008) for de novo gene prediction. We mapped the RNA-seq data to the assembled reference genome using TopHat v2.1.1 (Trapnell et al. 2012), followed by gene prediction using Cufflinks v2.1.1 (Trapnell et al. 2012). Finally, the predicted gene structures from the aforementioned three methods were merged into a nonredundant and complete gene model using MAKER v.1.0 (Holt and Yandell 2011).

We used BLAST v2.2.31 (Altschul et al. 1990) for gene function information and aligned the annotation results by using five protein databases, which included Swiss-Prot (Boeckmann et al. 2003), TrEMBL (Boeckmann et al. 2003), InterPro (Jones et al. 2014), KEGG (Kanehisa and Goto 2000) and GO (Ashburner et al. 2000).

For non-coding RNAs annotation, transfer RNAs (tRNA) were predicted using tRNAscan-SE v1.3.1 (Lowe and Eddy 1997). The BLASTN algorithm was used to align the Ribosomal RNAs (rRNA) template sequences from the Rfam database (Griffiths-Jones et al. 2005) against the genome. Other non-coding RNAs, such as miRNAs and snRNAs, were predicted by homology searches against the Rfam database using INFERNAL software (Nawrocki et al. 2009).

### Genome evolution analysis

We conducted gene family clustering analysis using genes from the whole-genome sequences of 28 species, including 16 species from the Fagaceae. These Fagaceae species comprised five *Castanopsis* species (*C. carlesii*, *C. chinensis*, *C. eyrei*, *C. hystrix*, *C. tibetana*), five *Castanea* species (*Ca. crenata*, *Ca. dentata*, *Ca. henryi*, *Ca. mollissima*, *Ca. sativa*), five *Quercus* species (*Q. robur*, *Q. mongolica*, *Q. acutissima*, *Q. suber*, *Q. lobata*), and one *Fagus* species (*F. sylvatica*) (Table S18). We constructed the protein datasets for these genomes and then used BLASTP (E-value of 1E-5, similarity threshold of 30%, and coverage threshold of 50%) to align the protein datasets (Kent 2002). OrthoFinder v2.2.6 (Emms and Kelly 2015) was used to identify orthologous groups among species. GO and KEGG enrichment analyses were performed using the R package clusterProfiler (Yu et al. 2012).

Furthermore, we obtained reliable single-copy orthologous groups by filtering out proteins less than 50 amino acids in length. Multisequence alignment of the amino acid sequences of single-copy orthologous groups was performed using MUSCLE v3.8.31 (Edgar 2004). Single-copy orthologous groups were connected to a supermatrix, which was used to construct a Bayesian phylogenetic tree. In Bayesian phylogenetic analysis, the divergence times were inferred using the MCMCTREE program of the PAML package v4.7 (Yang 2007). The gene family expansion and contraction in each tree node were inferred using CAFE 4.2 (De Bie et al. 2006). The CAFE uses a stochastic birth–death model to simulate the gains and losses of gene families in a phylogenetic process and infer the size of gene families of ancestral species. The expansion or contraction of the gene family of a target species is defined by comparing the increase or decrease in the number of gene family members between the target species and its ancestors. The Markov chain Monte Carlo (MCMC) process of PAML MCMCTREE was run 1,500,000 times, with the sample frequency set to 150 after a burn-in of 500,000 iterations, and the other parameters were set as default. We performed two independent runs to confirm that the different runs produced similar results. The selection of calibration time refers to the TimeTree database (www.timetree.org) (Kumar et al. 2022), and the selection was as follows: *P. persica*–*J. regia* divergence (89–113 Mya), *P. persica*–*P. trichocarpa* divergence (101–131 Mya), *P. trichocarpa–V. vinifera* divergence (107–135 Mya), *A. thaliana–P. trichocarpa* divergence (97–117 Mya), and monocotyledon–dicotyledon divergence (140–175 Mya). We also used fossil records of *Castanopsis* (52 Mya) (Wilf et al. 2019) and Fagaceae (81–82 Mya) (Grímsson et al. 2016) for the calibration.

### Collinearity analysis and whole-genome duplication

JCVI v1.2.7 (Tang et al. 2024) was used to analyze the protein sequences of *C. carlesii* and *Ca. henryi* and related species to obtain gene pairs in their collinear regions using default parameters.

To estimate the polyploidization events, the distribution of *Ks* in the genomes of *C. carlesii* and *Ca. henryi* and *P. persica* were identified. Protein sequence self-alignment was performed using Diamond in the genomes of the species. Mutually optimal alignments were extracted from the alignment results. The *Ks* values for the genome of *C. carlesii*, *Ca. henryi*, and *P. persica* were calculated using COMDEML in the PAML package v4.7 (Yang 2007).

### Transcriptomic assembly and expression analysis

Raw reads were obtained by Illumina HiSeq sequencing and filtered using SOAPnuke v1.5.3 (Chen et al. 2018). HISAT2 v2.1.0 (Kim et al. 2015) were used to map clean sequences of *C. carlesii* and *Ca. henryi* reference genomes. RSEM v1.2.8 (Li and Dewey 2011) was used to calculate the gene expression level of each sample.

### Gene family analysis

HMM profiles of MADS (PF00319) were obtained from Pfam (http://pfam.xfam.org/). MADS-box candidate gene proteins were searched using HMMER v3.2.1 (Eddy 2011) (with default parameters) and InterProScan (Zdobnov and Apweiler 2001). For genes related to sucrose metabolism, starch synthesis, and starch degradation, the related gene protein sequences were downloaded from TAIR (https://www.arabidopsis.org/) as a reference and BLASTP was used to search for related gene protein sequences. The predicted genes were manually inspected, and their domains were identified using SMART (Letunic et al. 2015). MEGA v7.0 (Kumar et al. 2016) was used to align candidate gene families and a phylogenetic tree was constructed using the neighbor-joining method with 1000 bootstrap replicates.

In order to identify the NLR genes, we employed the Resistify v0.1.1 (Smith et al. 2024). Firstly, Resistify conducts a hmm search on the input protein sequence to identify sequences containing coiled-coil (CC), Resistance to Powdery Mildew 8-like (RPW8), Toll/Interleukin-1 receptor (TIR), and NB-ARC domains. Subsequently, use the NLRexpress model to search for NLR related motifs. Then further re annotate CC, TIR, and Leucine-Rich-Repeat (LRR), and extract the protein sequence of the NB-ARC domain.

Gene protein sequences were aligned using the default parameters of the MUSCLE v3.8.31 (Edgar 2004) in MEGA v7.0 (Kumar et al. 2016) software, and phylogenetic analysis was performed by the Neighbor-Joining method with 1000 bootstraps. To analyze the chromosomal location of genes, we used the TBtools v2.136 (Chen et al. 2020) software to obtain information on the location of genes, and then plotted the gene locations.

### Sucrose and starch content determination

The collected fruit samples were separated into cupule, fruit skin, and seed coat, with the seed endosperm extracted. The endosperm was steamed at 105 °C in an oven for 15 min, dried at 65 °C to a constant weight, ground into powder, and used to determine starch and sucrose content. Three biological replicates were performed. Sucrose content was measured using a plant sucrose detection kit (BC2460, Solarbio, Beijing) and visible spectrophotometry, with approximately 50 mg of powdered sample. Starch content was analyzed via dual-wavelength spectrophotometry using a UV-2102C spectrophotometer, measuring amylopectin at 535 nm and amylose at 630 nm, with the same sample size.

### Paraffin section

Place the material in Formalin-Acetic Acid-Alcohol (FAA) fixative for 24 h, then dehydrate it by sequentially immersing it in graded ethanol solutions (50%, 70%, 85%, 90%, and 100%) at room temperature for 1 h each to remove water. Transfer the material into solutions of 100% ethanol mixed with xylene at ratios of 3:1, 1:1, and 1:3 (v/v), followed by pure xylene, for one hour per step to replace the alcohol with xylene and achieve transparency. Place the material in a xylene/paraffin (1:1, v/v) oven at 60 °C for 12 h, then embed the tissues by adding more paraffin regularly (per hour) until cultured in pure paraffin at 60 °C for 12 h. Finally, replace the paraffin three times every 3 h and vacuum infiltrate at 60 °C for 30 min. Embed the tissue in paraffin wax, then section the sample using a Leica RM2235 microtome to produce 8 μm-thick wax strips. The sections stained with Safranin O (1%)/Fast green (0.5%), sealed by neutral balsam. Finally, fix the sections, cover them with a coverslip, and observe the sections under a light microscope (ECLIPSE Ci-L; Nikon).

## Supplementary Information

Additional file 1: Fig. S1. Genome size and heterozygosity of *C. carlesii* and *Ca. henryi* estimation using 17 K-mer distribution. Fig. S2. Interchromosomal Hi-C contact map of (a) *C. carlesii* and (b) *Ca. henryi* genome. Fig. S3. Evaluation of genome assemblies by LTR Assembly Index (LAI). Fig. S4. Insertion time distribution of long terminal repeat retrotransposons (LTR-RTs) in the genomes of nine Fagaceae species. Fig. S5. Phylogenetic tree of MADS-box genes from *C. carlesii*, *Ca. henryi*, *A. thaliana*, and *P. persica*. Fig. S6. Phylogenetic tree of (a) *AG* subfamily genes and (b) *SVP* genes from *C. carlesii*, *Ca. henryi*, *A. thaliana*, and *P. persica.* Fig. S7. Chromosome location of MADS-box genes in (a) *C. carlesii* and (b) *Ca. henryi*. *SVP* genes are highlighted in red. Fig. S8. Microstructure observation of staminodes in female flowers of *C. carlesii.* Se, stamens. S, stigma. Fig. S9. Morphological characteristics of seven different developmental stages of the fruit of *C. carlesii.* Fig. S10. Dynamic changes of sucrose content and starch content of *C. carlesii* fruits at seven different development stages.

Additional file 2: Table S1. Assembled statistics of *C. carlesii* and *Ca. henryi.* Table S2. The chromosome length of *C. carlesii* and *Ca. henryi.* Table S3. Benchmarking Universal Single-Copy Orthologs (BUSCO) evaluation of *C. carlesii* and *Ca. henryi* genome assembly. Table S4. Evaluation of genome assemblies by LTR Assembly Index (LAI). The LAI scores distribution of *C. carlesii* and *Ca. henryi* 12 chromosomes. Table S5. Prediction of gene structures of *C. carlesii* and *Ca. henryi* genome. Table S6. Benchmarking Universal Single-Copy Orthologs (BUSCO) assessment of *C. carlesii* and *Ca. henryi* annotated genome. Table S7. Statistics on the function annotation of the *C. carlesii* and *Ca. henryi* genome. Table S8. Non-coding RNA annotation results of *C. carlesii* and *Ca. henryi* genome. Table S9. Statistic of repeat sequence in *C. carlesii* and *Ca. henryi* genome. Table S10. Gene-clustering statistics for 28 species. Table S11. Gene ontology (GO) and Kyoto Encyclopedia of Genes and Genomes (KEGG) enrichment result of unique gene of Fagaceae. Table S12. Gene ontology (GO) and Kyoto Encyclopedia of Genes and Genomes (KEGG) enrichment result of significant expansion of *Castanopsis* and *Castanea* gene families. Table S13. Genome-wide collinearity analysis statistical results. Table S14. List of MADS-box genes identified in *A. thaliana*, *P. persica*, *C. carlesii*, *C. tibetana*, *Ca. henryi*, *Ca. mollissima*, *Q. acutissima*, *Q. mongolica*, and *F. sylvatica*. Table S15. Sucrose-starch metabolism related gene identified in *C. carlesii* genome. Table S16. Sucrose content and starch content of *C. carlesii* fruits at different development stages. Table S17. Summary of nucleotide-binding leucine-rich repeat (NLR) genes identification results for Fagaceae. Table S18. List of species sources used in this study.

## Data Availability

The genome sequencing data and transcriptome sequencing data have been deposited at the National Genomics Data Center (NGDC, https://ngdc.cncb.ac.cn) under accession number PRJCA021809 and PRJCA021810.

## Abbreviations

*AG*: *AGAMOUS*
*AGL11*: *AGAMOUS-LIKE11*
*AGL24*: *AGAMOUS-LIKE24*
BUSCO: Benchmarking Universal Single-Copy Orthologs
CC: Coiled-coil
*DAM*: *Dormancy-associated MADS-box*
F6P: Fructose-6-phosphate
Fru: Fructose
G1P: Glucose-1-phosphate
Glu: Glucose
GO: Gene ontology
INV: Invertase
KEGG: Kyoto Encyclopedia of Genes and Genome
*Ks*: Synonymous substitutions per synonymous site
LAI: LTR Assembly Index
LRR: Leucine-Rich-Repeat
LTR-RT: Long terminal repeat retrotransposons
MCMC: Markov chain Monte Carlo
NB-ARC: Nucleotide binding domain
NLR: Nucleotide-binding leucine-rich repeat
RPW8: Powdery Mildew 8-like
SMRT: Single-molecule real-time
*STK*: *SEEDSTICK*
SUS: Sucrose synthase
*SVP*: *SHORT VEGETATIVE PHASE*
TEs: Transposable elements
TIR: Toll/Interleukin-1 receptor
UDP: Uridine diphosphate
UDPG: UDP-glucose
WGD: Whole genome duplication
