# Supplementary material for: Genomic insights into Castanopsis carlesii and Castanea henryi: flower and fruit development and evolution of NLR genes in the beech-oak family
Source: Mol Hortic. 2025 Jun 4;5:33. doi: 10.1186/s43897-025-00152-4 (PMC12135283; doi:10.1186/s43897-025-00152-4)
Supplement: Supplementary file 1 — Additional file 1: Fig. S1. Genome size and heterozygosity of C. carlesii and Ca. henryi estimation using 17 K-mer distribution. Fig. S2. Interchromosomal Hi-C contact map of (a) C. carlesii and (b) Ca. henryi genome. Fig. S3. Evaluation of genome assemblies by LTR Assembly Index (LAI). Fig. S4. Insertion time distribution of long terminal repeat retrotransposons (LTR-RTs) in the genomes of nine Fagaceae species. Fig. S5. Phylogenetic tree of MADS-box genes from C. carlesii, Ca. henryi, A. thaliana, and P. persica. Fig. S6. Phylogenetic tree of (a) AG subfamily genes and (b) SVP genes from C. carlesii, Ca. henryi, A. thaliana, and P. persica. Fig. S7. Chromosome location of MADS-box genes in (a) C. carlesii and (b) Ca. henryi. SVP genes are highlighted in red. Fig. S8. Microstructure observation of staminodes in female flowers of C. carlesii. Se, stamens. S, stigma. Fig. S9. Morphological characteristics of seven different developmental stages of the fruit of C. carlesii. Fig. S10. Dynamic changes of sucrose content and starch content of C. carlesii fruits at seven different development stages. [file 43897_2025_152_MOESM1_ESM.docx]

**Additional file 1**

**Content**

[**Fig. S1** Genome size and heterozygosity of *C. carlesii* and *Ca. henryi* estimation using 17 *K*-mer distribution. 2](#_Toc191674045)

[**Fig. S2** Interchromosomal Hi-C contact map of (a) *C. carlesii* and (b) *Ca. henryi* genome. 3](#_Toc191674046)

[**Fig. S3** Evaluation of genome assemblies by LTR Assembly Index (LAI). 4](#_Toc191674047)

[**Fig. S4** Insertion time distribution of long terminal repeats (LTRs) in the genomes of nine Fagaceae species*.* 5](#_Toc191674048)

[**Fig. S5** Phylogenetic tree of MADS-box genes from *C. carlesii*, *Ca. henryi*, *A. thaliana*, and *P. persica.* 6](#_Toc191674049)

[**Fig. S6** Phylogenetic tree of (a) *AG* subfamily genes and (b) *SVP* genes from *C. carlesii*, *Ca. henryi*, *A. thaliana*, and *P. persica.* 7](#_Toc191674050)

[**Fig. S7** Chromosome location of MADS-box genes in (a) *C. carlesii* and (b) *Ca. henryi*. *SVP* genes are highlighted in red. 8](#_Toc191674051)

[**Fig. S8** Microstructure observation of staminodes in female flowers of *C. carlesii.* Se, stamens. S, stigma. 9](#_Toc191674052)

[**Fig. S9** Morphological characteristics of seven different developmental stages of the fruit of *C. carlesii.* 10](#_Toc191674053)

[**Fig. S10** Dynamic changes of sucrose content and starch content of *C. carlesii* fruits at seven different development stages. 11](#_Toc191674054)





**Fig. S1** Genome size and heterozygosity of *C. carlesii* and *Ca. henryi* estimation using 17 *K*-mer distribution. (a) *K*-mer analysis shows that the genome size of *C. carlesii* is about 899.76 Mb with heterozygosity of 1.59%. (b) *K*-mer analysis shows that the genome size of *Ca. henryi* is about 755.49 Mb with heterozygosity of 2.52%.


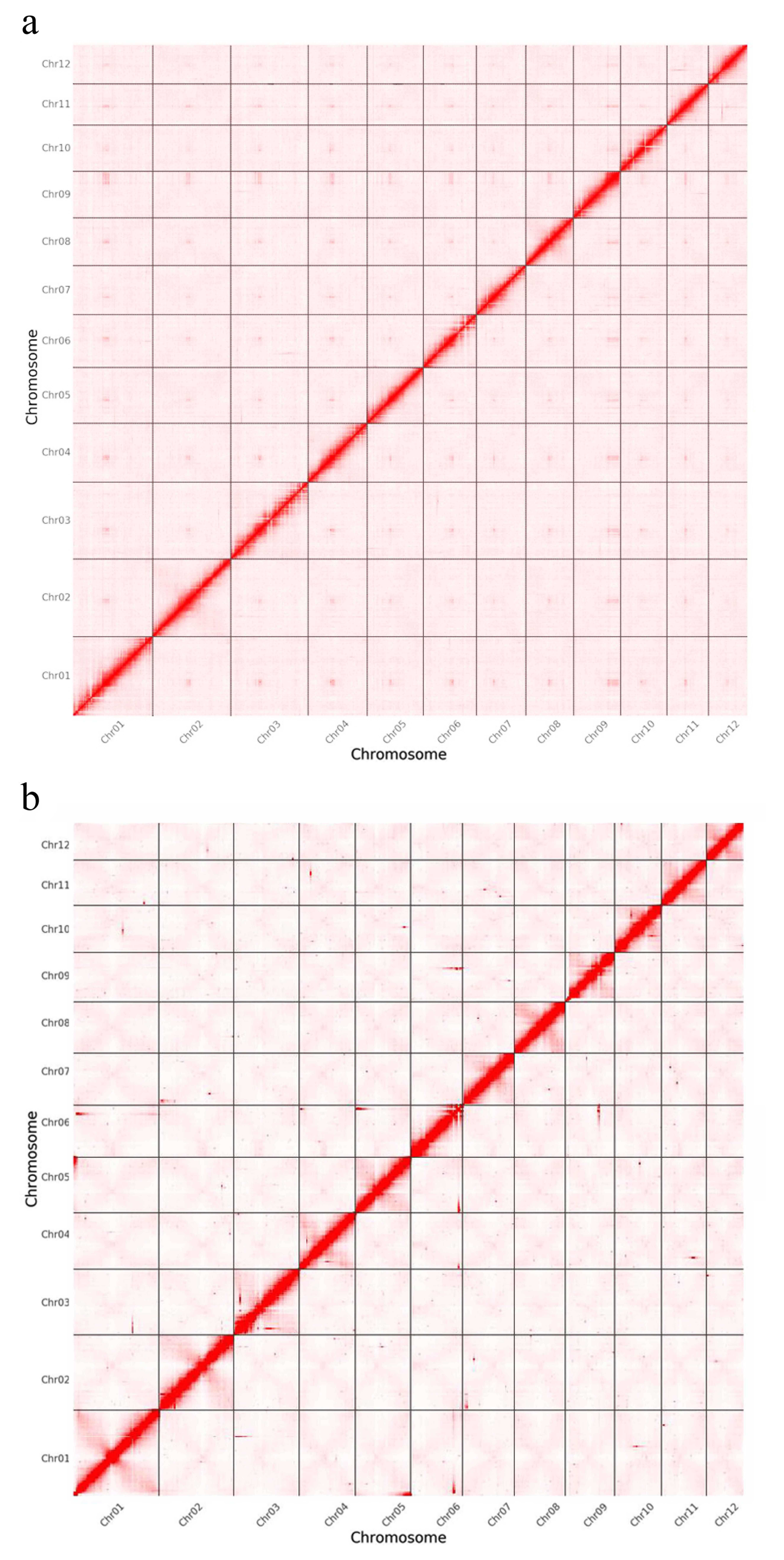


**Fig. S2** Interchromosomal Hi-C contact map of (a) *C. carlesii* and (b) *Ca. henryi* genome. The frequency of Hi-C interaction links was represented by the colour ranging from white (low) to red (high). Most interactions were observed within the chromosomes.


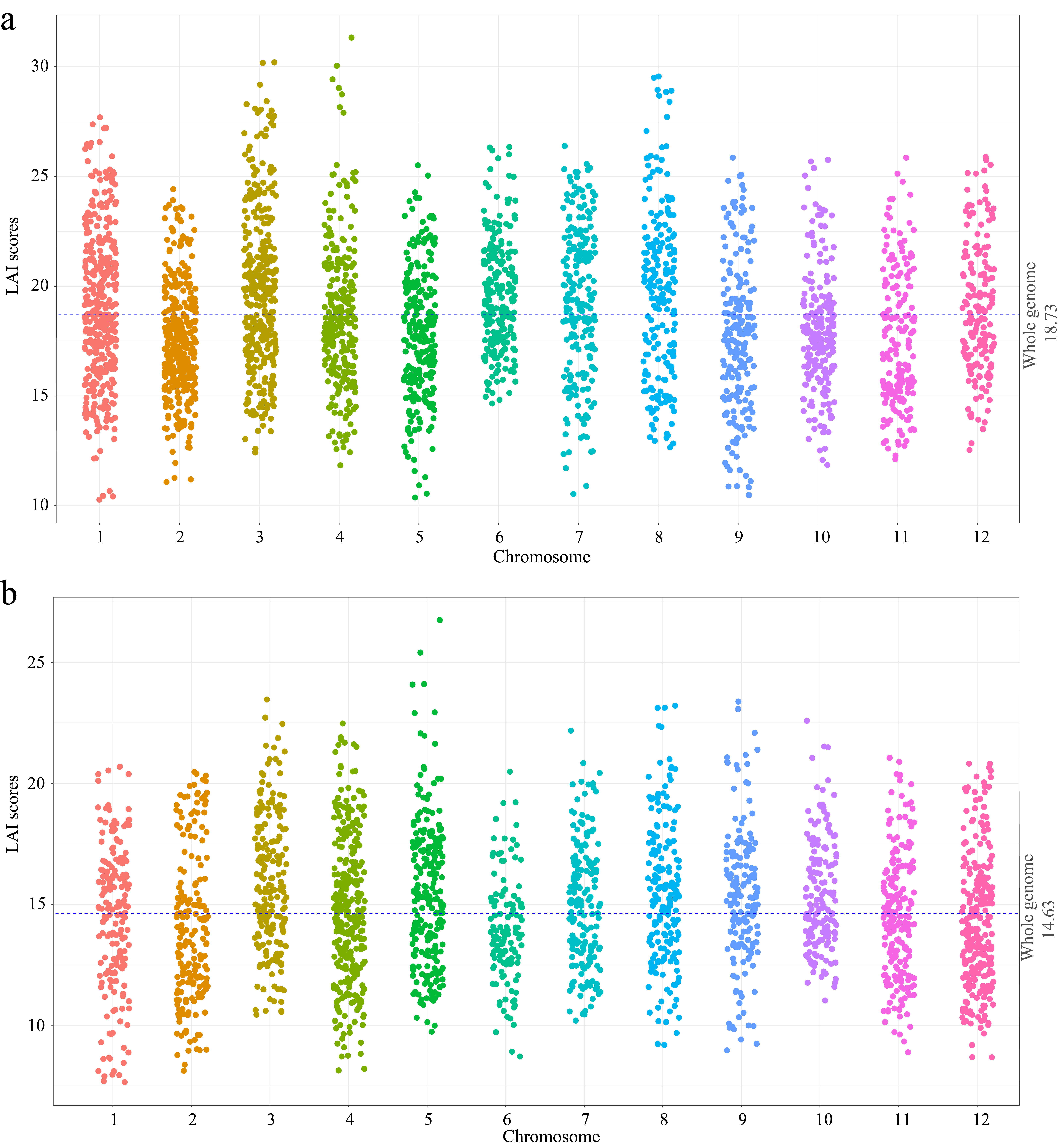


**Fig. S3** Evaluation of genome assemblies by LTR Assembly Index (LAI). (a) The LAI scores distribution of *C. carlesii* 12 chromosomes. (b) The LAI scores distribution of *Ca. henryi* 12 chromosomes. The x-axes show the chromosomes of each genome. LAI scores, represented by the dots, were calculated using 3 Mb sliding windows. The blue dashed line indicates the whole genome average of LAI score.


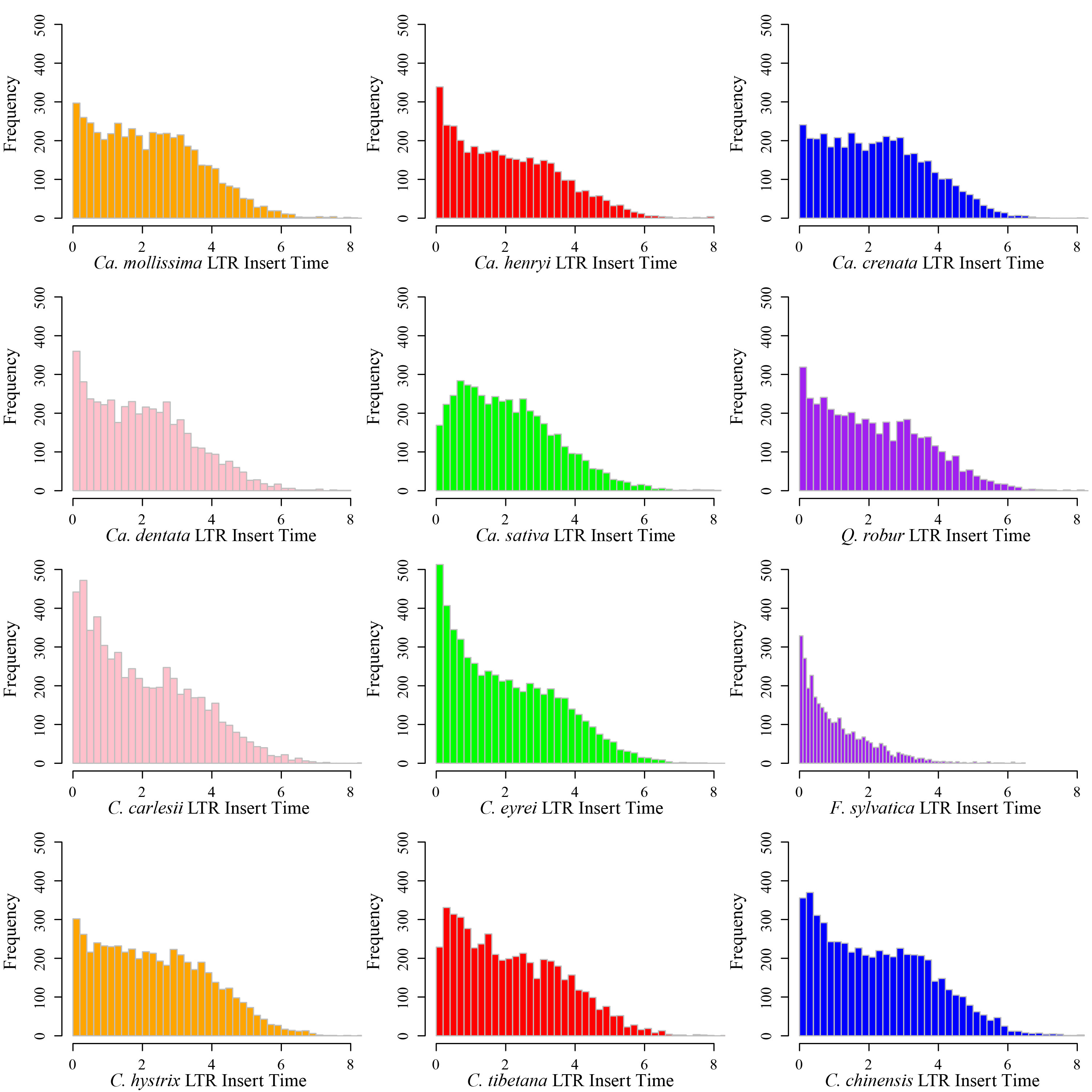


**Fig. S4** Insertion time distribution of long terminal repeats (LTRs) in the genomes of nine Fagaceae species, including five *Castanea* species (*Ca. mollissima*, *Ca. henryi*, *Ca. crenata*, *Ca. dentata*, and *Ca. sativa*), five *Castanopsis* species (*C. carlesii*, *C. eyrei*, *C. hystrix*, *C. tibetana*, and *C. chinensis*), *Q. acutissima*, and *F. sylvatica.*

**Fig. S5** Phylogenetic tree of MADS-box genes from *C. carlesii*, *Ca. henryi*, *A. thaliana*, and *P. persica.*


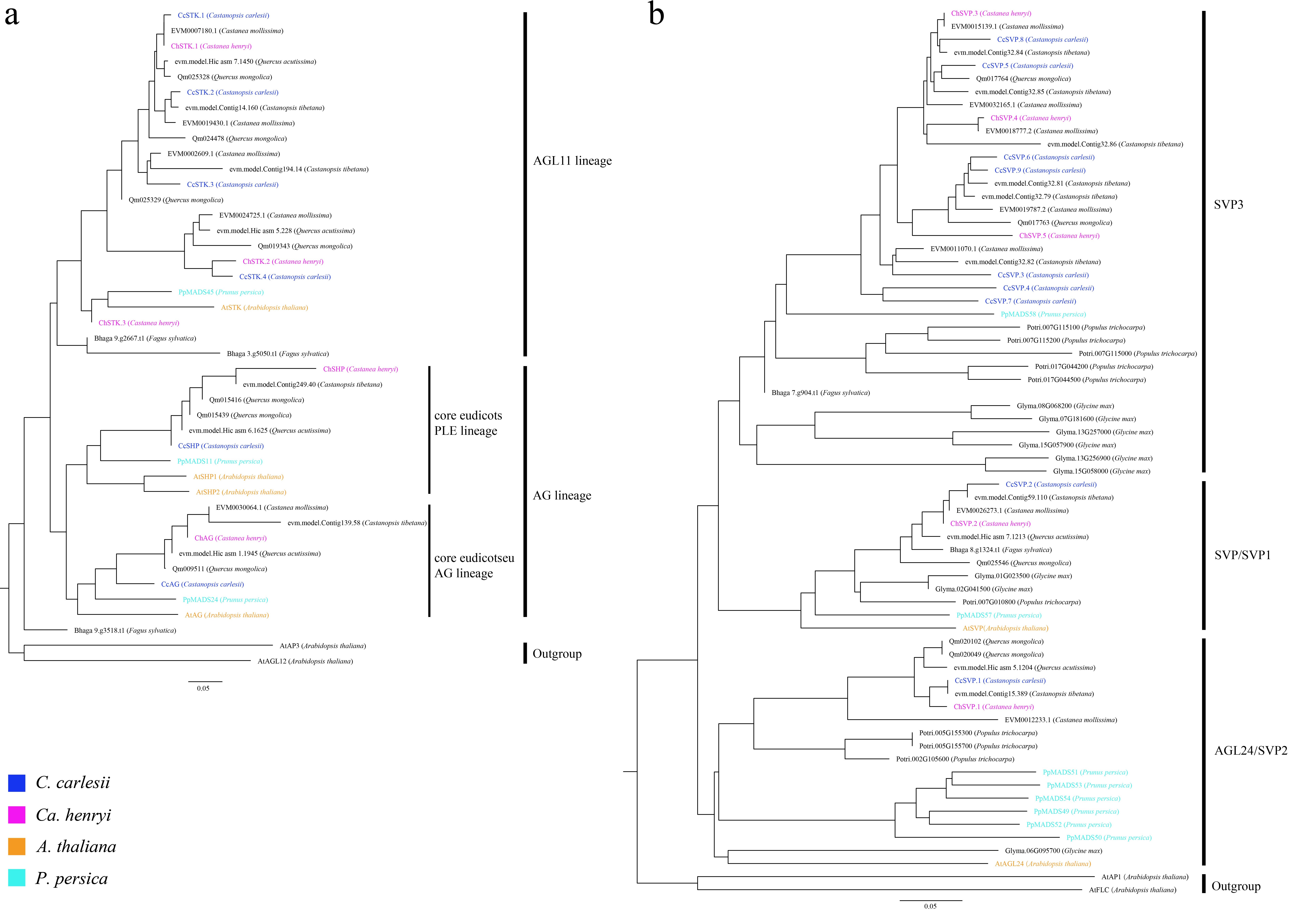


**Fig. S6** Phylogenetic tree of (a) *AG* subfamily genes and (b) *SVP* genes from *C. carlesii*, *Ca. henryi*, *A. thaliana*, and *P. persica.*





**Fig. S7** Chromosome location of MADS-box genes in (a) *C. carlesii* and (b) *Ca. henryi*. *SVP* genes are highlighted in red.


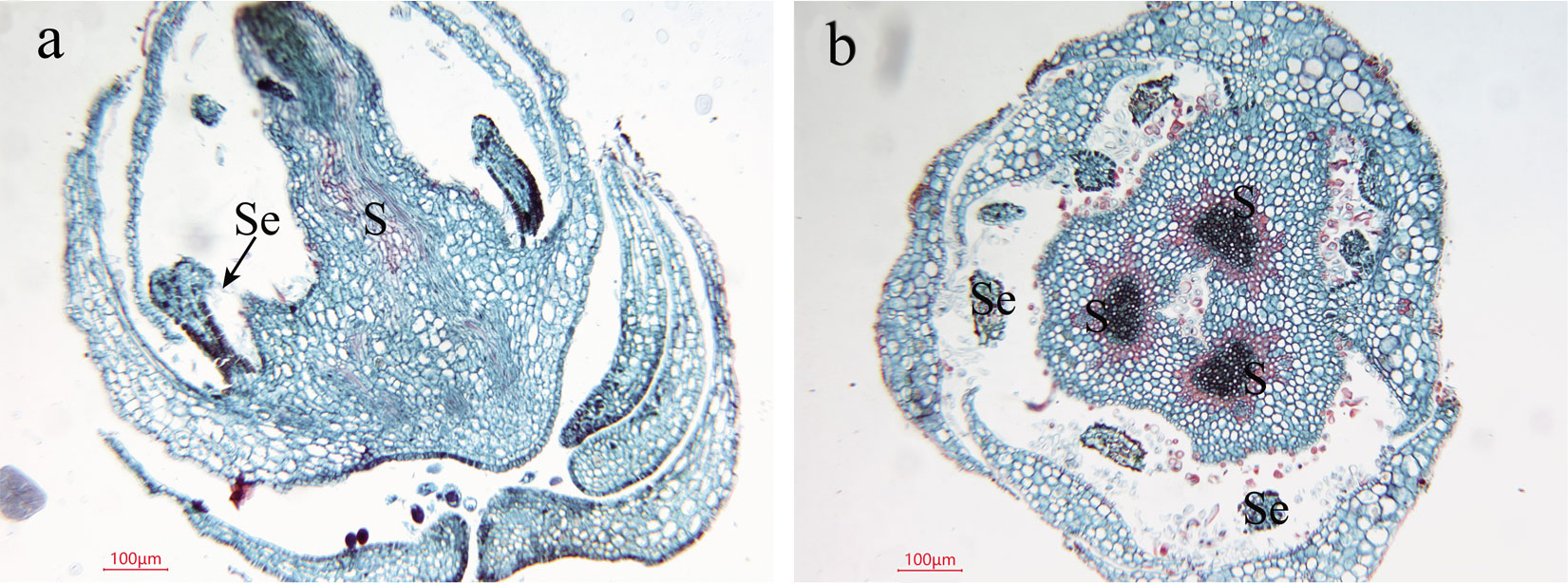


**Fig. S8** Microstructure observation of staminodes in female flowers of *C. carlesii.* Se, stamens. S, stigma. (a) The longitudinal sections of female flowers showing the developing stamens, and stigma. (b) The transverse section of female flower showing staminode and stigma.


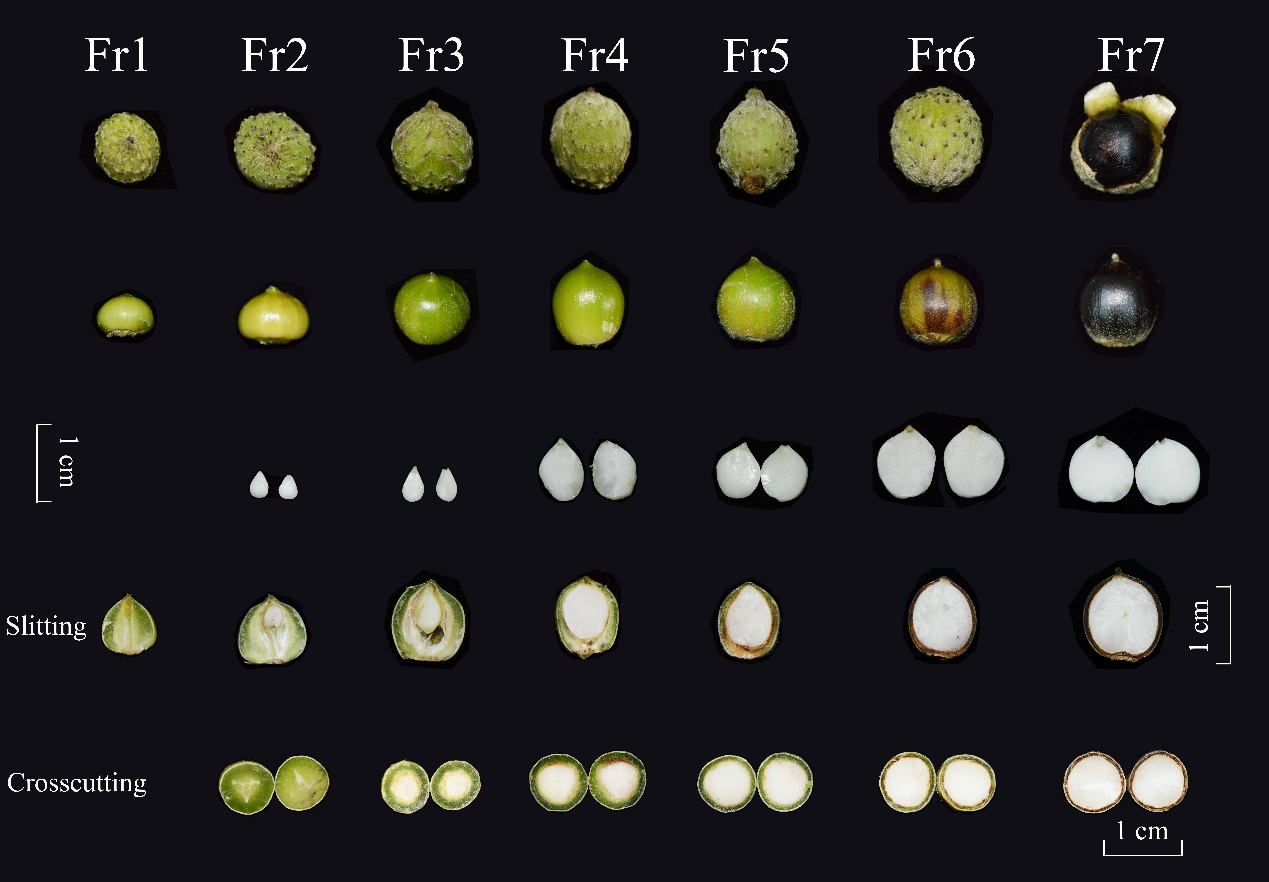


**Fig. S9** Morphological characteristics of seven different developmental stages of the fruit of *C. carlesii.*


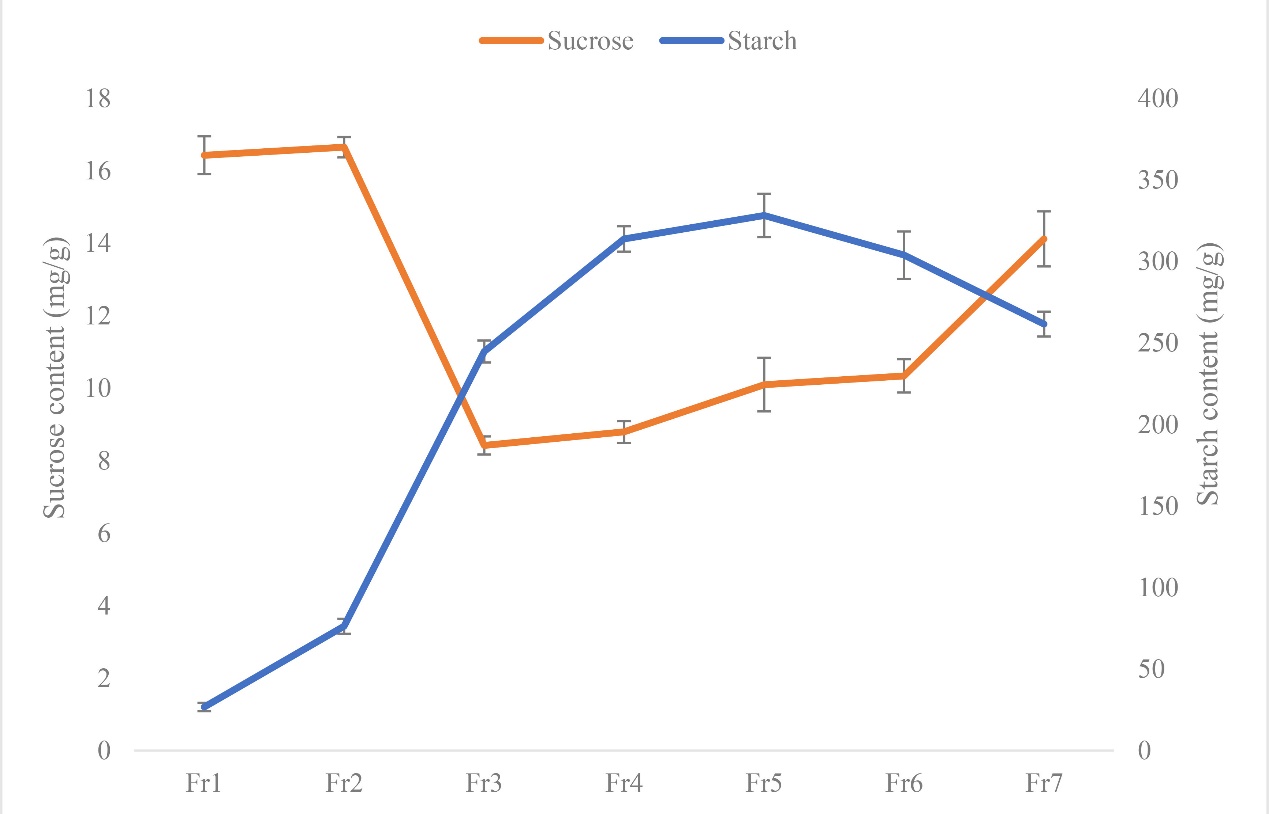


**Fig. S10** Dynamic changes of sucrose content and starch content of *C. carlesii* fruits at seven different development stages. Orange line represents sucrose content, and blue line represents starch content.
